# Type 2 diabetes in people living with HIV: epidemiology, mechanisms, sex differences and early-life determinants

**DOI:** 10.3389/fendo.2026.1734023

**Published:** 2026-02-10

**Authors:** Raquel Moreno-Lopez, Beatriz Lazaro-Martin, Cristina Díez, Maria Luisa Navarro-Gomez, Laura Tarancon-Diez

**Affiliations:** 1 Grupo de Infecciones en la Población Pediátrica, Health Research Institute Gregorio Marañón (IiSGM), Madrid, Spain; 2 Centro de Investigación Biomédica en Red de Enfermedades Infecciosas (CIBERINFEC), Instituto de Salud Carlos III (ISCIII), Madrid, Spain; 3 Unidad de Enfermedades Infecciosas/VIH, Hospital General Universitario Gregorio Marañón, Instituto de Investigación Sanitaria Gregorio Marañón (IiSGM), Madrid, Spain; 4 Servicio de Pediatría, Hospital General Universitario Gregorio Marañón, Madrid, Spain; 5 Departamento de Pediatría, Universidad Complutense de Madrid (UCM), Madrid, Spain

**Keywords:** adolescents and young adults, antiretroviral therapy, HIV infection, insulin resistance, metabolic syndrome, perinatal HIV infection, sex differences, type 2 diabetes mellitus

## Abstract

The growing coexistence of HIV infection and type 2 diabetes mellitus (T2DM) represents a major clinical challenge in the antiretroviral therapy (ART) era. Improved survival of people living with HIV (PLHIV) has unveiled an increasing burden of metabolic disorders, with T2DM emerging as a leading comorbidity linked to chronic inflammation, adipose dysfunction, hepatic steatosis, and gut–liver axis disruption. Epidemiological evidence indicates that PLHIV develop diabetes at younger ages and with greater cardiometabolic complications than the general population. Among adolescents and young adults with perinatally acquired HIV, lifelong ART exposure and early-life immune activation accelerate insulin resistance and β-cell stress, predisposing to early-onset T2DM. Sex differences further modulate this risk, as women with HIV exhibit disproportionate weight gain, altered fat distribution, and heightened inflammatory responses under specific ART regimens. The convergence of immunometabolic imbalance, hormonal factors, and social determinants creates a distinct pathophysiological landscape demanding tailored prevention and management strategies. Novel incretin-based and amylin therapies hold promise to address both dysglycemia and obesity, though data in PLHIV remain limited. Recognizing diabetes as a central and multifactorial complication of HIV is crucial to optimize long-term care, reduce cardiovascular and hepatic comorbidities, and improve quality of life across the HIV lifespan.

## 1 Introduction

The widespread use of antiretroviral therapy (ART) has transformed HIV infection into a chronic, manageable condition, allowing people living with HIV (PLHIV) to achieve near-normal life expectancy (1, 2). As survival improves, the burden of non-AIDS comorbidities has become increasingly prominent, with type 2 diabetes mellitus (T2DM) emerging as a major contributor to long-term morbidity and mortality in this population (3, 4).

The epidemiology of T2DM in PLHIV is shaped by a complex interplay of traditional metabolic risk factors, HIV-related inflammation and immune activation, and the long-term effects of ART (5–7). These risks are not evenly distributed: women often show disproportionate ART-associated weight gain, while adolescents and young adults with perinatally acquired HIV face unique vulnerabilities related to lifelong infection and treatment during critical developmental stages.

Given these multifactorial drivers, early diagnosis is central to preventing progression to overt diabetes and reducing the risk of complications such as cardiovascular, renal, and neurocognitive disease. Current guidelines advocate for annual screening; however, optimal screening strategies in PLHIV remain debated, as conventional diagnostic tools may perform inconsistently in this population and there is ongoing discussion regarding which glycemic markers are the most effective. This highlights the need not only for systematic and repeated screening but also for the identification of predictive biomarkers that can capture the unique pathophysiological mechanisms linking HIV and T2DM.

In this context, emerging research on immuno-inflammatory markers, metabolomic profiles, and gut microbiota signatures provides promising avenues to refine risk stratification and guide individualized prevention strategies. Strengthening these efforts will be key to advancing precision medicine approaches capable of addressing the dual challenge of HIV and diabetes.

This review synthesizes current evidence on the epidemiology, underlying drivers, and clinical implications of T2DM in PLHIV, with a focus on sex- and age-related disparities. We further examine the challenges in diagnosis and treatment, and highlight emerging opportunities, including predictive biomarkers, to guide future research and improve clinical management of T2DM in this population.

## 2 Fundamentals of type 2 diabetes mellitus: pathogenesis, diagnosis, and management

Type 2 diabetes mellitus (T2DM) is a complex metabolic disorder that results from the combined effects of insulin resistance and progressive pancreatic β-cell dysfunction. At the level of insulin-sensitive tissues, several abnormalities converge. In the liver, impaired insulin signaling promotes excessive gluconeogenesis and inappropriate glucose output, while in skeletal muscle the translocation of glucose transporter 4 (GLUT4) is reduced, leading to diminished glucose uptake. In adipose tissue, accelerated lipolysis increases circulating free fatty acids, which contribute to ectopic lipid accumulation in liver and muscle and perpetuate insulin resistance and lipotoxicity (8). Among PLHIV, it has been observed that the long chain fatty acid eicosenoate inhibits T-cell function (9), and their insulin resistance profiles are characterized by lower concentrations of circulating medium and long-chain acylcarnitines (10). Chronic exposure to hyperglycemia and elevated free fatty acids produces glucotoxicity and lipotoxicity, together with oxidative and endoplasmic reticulum stress and deposition of islet amyloid, ultimately resulting in progressive β-cell failure and declining insulin secretion (11, 12).

Similarly to what can be observed in ART-treated PLHIV, low-grade chronic inflammation has emerged as a central feature of T2DM pathogenesis. Adipose tissue expansion is accompanied by recruitment of pro-inflammatory macrophages and dysregulated secretion of adipokines, including increased tumor necrosis factor-α (TNF-α) and interleukin-6 (IL-6) and reduced adiponectin, all of which exacerbate systemic insulin resistance (13). In addition, defects in the incretin axis, characterized by diminished secretion and action of glucagon-like peptide 1 (GLP-1) and glucose-dependent insulinotropic polypeptide (GIP), contribute to impaired postprandial insulin secretion and inappropriate glucagon responses (14). Other factors, including non-alcoholic fatty liver disease, alterations in the gut microbiome, mitochondrial dysfunction, circadian disruption and sleep disorders, have been implicated as modulators of the metabolic milieu (15). Importantly, T2DM is not a uniform disease but a heterogeneous condition. Subgroup analyses have identified clusters ranging from obesity-driven insulin resistance to more insulin-deficient lean phenotypes, and youth-onset diabetes has been shown to follow a particularly aggressive course (16).

Diagnosis of T2DM relies on biochemical criteria established by international guidelines. According to the American Diabetes Association (17) and the World Health Organization (18), diabetes may be diagnosed by a fasting plasma glucose level of at least 126 mg/dL (7.0 mmol/L), a two-hour plasma glucose of at least 200 mg/dL (11.1 mmol/L) during a 75-g oral glucose tolerance test, a glycated hemoglobin (HbA1c) of 6.5% (48 mmol/mol) or higher, or a random plasma glucose of at least 200 mg/dL (11.1 mmol/L) in the presence of classic symptoms of hyperglycemia. Prediabetes is defined by intermediate thresholds, including fasting plasma glucose of 100–125 mg/dL, a two-hour value of 140–199 mg/dL, or an HbA1c between 5.7% and 6.4%. The oral glucose tolerance test remains the most sensitive diagnostic tool, particularly for identifying early postprandial dysglycemia, while HbA1c measurement may be limited in conditions that affect red cell turnover or in populations with hemoglobinopathies (18).

The treatment of T2DM aims not only to achieve and maintain durable glycemic control but also to prevent or delay complications, reduce cardiovascular and renal risk and improve quality of life. Lifestyle modification represents the foundation of management and includes structured nutritional plans, regular physical activity and cessation of smoking (19). When lifestyle measures are insufficient, pharmacological treatment is required. Metformin remains the recommended first-line therapy due to its efficacy, safety, low cost, and favorable effects on weight and cardiovascular risk profile (20). In recent years, the therapeutic landscape has expanded with the introduction of agents that provide benefits beyond glycemic control. Glucagon-like peptide 1 receptor agonists, such as liraglutide and semaglutide, and sodium-glucose cotransporter 2 inhibitors, such as empagliflozin and dapagliflozin, have demonstrated significant reductions in cardiovascular and renal outcomes in large randomized trials and are now prioritized in individuals with established cardiovascular disease, chronic kidney disease or heart failure (21–23). Other options include dipeptidyl peptidase 4 inhibitors and thiazolidinediones, while sulfonylureas and insulin remain effective but carry a higher risk of hypoglycemia and weight gain. In patients with obesity and inadequate control, metabolic or bariatric surgery has proven to be an effective intervention that can induce remission of diabetes in a substantial proportion of cases (24). Optimal management also encompasses comprehensive cardiovascular risk reduction, including blood pressure control, lipid-lowering therapy with statins according to cardiovascular risk, and antiplatelet therapy in selected high-risk individuals (17).

The development of T2DM is influenced by a range of risk factors. Non-modifiable determinants include age, family history, and genetic susceptibility, as well as high-risk ancestry such as South Asian, Hispanic/Latino, Black/African, Native American, and Pacific Islander backgrounds. Modifiable factors play a dominant role and include overweight and obesity, central adiposity, physical inactivity, unhealthy dietary patterns rich in refined carbohydrates and saturated fats, smoking, and excessive alcohol consumption (25). A number of clinical correlates further increase risk, including hypertension, atherogenic dyslipidemia, metabolic syndrome, non-alcoholic fatty liver disease, and obstructive sleep apnea. Moreover, several commonly used medications, such as glucocorticoids, certain antipsychotics, calcineurin inhibitors, and some immunosuppressants, can precipitate or exacerbate dysglycemia (17). In addition to biological and clinical factors, social determinants of health have emerged as critical drivers of both incidence and outcomes. Socioeconomic deprivation, food insecurity, health care disparities and psychosocial stressors strongly influence both the risk of developing T2DM and the ability to achieve adequate disease control (26).

## 3 Epidemiology of type 2 diabetes mellitus in people living with HIV

The risk and incidence of T2DM among people living with HIV (PLHIV) were already recognized in the early era of antiretroviral therapy (ART), with frequencies beyond what would be expected from traditional risk factors alone (27, 28). As ART evolved and regimens with lower metabolic toxicity became available, reported rates of T2DM varied more widely, with substantial differences across cohorts and geographical settings (29).

Subsequent systematic reviews highlighted this heterogeneity. Nansseu et al. (2018) reported that the prevalence of T2DM in PLHIV depends on the population studied, the diagnostic criteria applied, and the size and design of the cohorts (30). The review encompassed both single-center and multicenter studies, spanning regions such as sub-Saharan Africa, Asia, and Latin America. Higher prevalence estimates were generally observed in African cohorts, suggesting that the interplay of ART exposure, genetic predisposition, and lifestyle transitions may amplify diabetes risk in these populations. Importantly, the review also emphasized the high burden of prediabetes in PLHIV, underscoring the vulnerability of this group to early metabolic disturbances. Similarly, Patel et al. (2018) documented a prevalence range of 1.3% to 18% in low- and middle-income countries, reinforcing the notion that the epidemiology of T2DM in HIV is shaped by demographic, clinical, and environmental factors (31).

Building on this heterogeneous evidence, recent cohort and population-based studies provide further insights into the prevalence and incidence of T2DM among PLHIV across different settings. Several investigations in high-income countries have consistently reported an excess risk. Brown et al. (27) demonstrated a fourfold higher risk of diabetes in PLHIV, while Hernandez-Romieu et al. (32) found that T2DM may occur at younger ages and even in the absence of obesity. In Europe, a Swedish cohort of PLHIV aged ≥50 years showed that baseline prevalence of T2DM was nearly twice that of the general population, and that the incidence of new-onset T2DM during follow-up was threefold higher (33). Similar trends have been documented in the United States, where Tiozzo et al. (4) reported higher rates of diabetes and Spieler et al. (3) observed a 5.2% increase in prevalence over ten years. Studies from Brazil, Iran, and Spain further confirm elevated risks, particularly in older adults, with a marked burden among individuals over 50 years (34–36).

In contrast, several studies from Africa and Asia have not detected significant differences between PLHIV and their HIV-negative counterparts. Peer et al. (37) in South Africa and Ye et al. (38) in China reported comparable prevalence rates, while Magodoro et al. (39) even found higher diabetes prevalence among HIV-negative individuals (15.9%) compared with PLHIV (9%). Regional evidence further complicates the picture: prevalence was estimated at 8.6% among PLHIV in the Middle East and North Africa (40), while in Canada, Bratu et al. (41) observed rising diabetes incidence over time in the HIV-negative population but not among PLHIV. Importantly, in a South African cohort, more than half (58.6%) of PLHIV meeting diagnostic criteria for T2DM were previously undiagnosed, underscoring the gaps in detection and the need for systematic screening (42, 43).

The heterogeneity of findings across studies likely reflects differences in methodology, diagnostic thresholds, and screening practices, as well as demographic and clinical characteristics of the populations studied. Additional sources of variability include the type and duration of ART exposure, the ability to adequately match HIV-negative controls, and disparities in healthcare infrastructure and resource availability across regions. Furthermore, reliance on HbA1c for diagnosis may be problematic in PLHIV, as HIV-related factors can affect its accuracy.

Taken together, current evidence suggests that the burden of T2DM among PLHIV is substantial but unevenly distributed across populations. While excess risk is consistently observed in high-income settings and in older cohorts, findings from low- and middle-income regions are less uniform and may be influenced by underdiagnosis and differences in study design. This variability highlights the need for harmonized diagnostic criteria, systematic screening, and locally adapted surveillance strategies to better define and address the true burden of T2DM in PLHIV.

## 4 Drivers of type 2 diabetes in people living with HIV: traditional and HIV-related factors

T2DM in PLHIV arises from the convergence of traditional metabolic determinants and factors directly related to HIV infection and its treatment. While aging, obesity, and unhealthy lifestyles remain central contributors, HIV-associated inflammation, lipodystrophy, and the long-term effects of ART add unique layers of risk. Understanding this multifactorial landscape is essential for tailoring prevention and management strategies in this population.

### 4.1 Traditional drivers

Traditional metabolic risk factors play a major role in the development of T2DM among PLHIV, mirroring patterns observed in the general population. Age, family history, obesity, and lifestyle determinants such as diet, sedentary behavior, alcohol consumption, and smoking have been repeatedly associated with increased diabetes risk (44–48). The D:A:D (Data Collection on Adverse Events of Anti-HIV Drugs) cohort, a large international prospective study initiated in 1999 to monitor long-term toxicities of ART, showed that elevated body mass index (BMI) predicted both T2DM and serious non-AIDS comorbidities (49). Moreover, weight gain following ART initiation has been linked to incident diabetes, reinforcing obesity as a critical pathway (50). Prediabetes is also highly prevalent in older PLHIV and strongly predicts progression to overt diabetes (51). The coexistence of metabolic abnormalities such as hyperglycemia, central obesity, hypertension, and atherogenic dyslipidemia further magnifies susceptibility, resembling the clustering of risk factors in the metabolic syndrome, which has been seen to have a relatively higher global magnitude among ART-exposed PLHIV than ART naïve groups (52).

### 4.2 HIV-related drivers

Beyond traditional risk factors, HIV infection itself contributes to diabetes risk through mechanisms of persistent immune activation, inflammation, and adipose tissue dysfunction. Even under effective ART, many PLHIV display elevated levels of inflammatory cytokines, including IL-6 and TNF-α, which impair insulin signaling and promote resistance to glucose uptake (6). Long-term exposure to HIV and ART has also been associated with lipodystrophy, characterized by abnormal fat redistribution, mitochondrial toxicity, and altered adipokine secretion, all of which compromise insulin sensitivity (6, 53). Additionally, advanced disease markers, such as low nadir CD4 counts and episodes of uncontrolled viremia, have been linked to insulin resistance and diabetes incidence, suggesting that prior immunodeficiency leaves a lasting metabolic imprint (54).

### 4.3 ART-related drivers

Since the introduction of ART, metabolic complications have been recognized as important contributors to T2DM risk in PLHIV. Early regimens, particularly those initiated before 1999, were consistently associated with increased diabetes incidence (55). First-generation protease inhibitors (PIs) reduced GLUT-4-mediated glucose transport and induced insulin resistance (56, 57), while thymidine analogue nucleoside reverse transcriptase inhibitors (NRTIs) were linked to insulin resistance and incident T2DM, with risk persisting even after drug discontinuation (54, 58–60). Older non-nucleoside reverse transcriptase inhibitors (NNRTIs) were not without risk either, as efavirenz exposure has been associated with elevated diabetes incidence (61). At that time, combination regimens were commonly referred to as highly active antiretroviral therapy (HAART), denoting the use of at least three drugs from two different classes. These early findings were consistent with data from the HAART era, where Samaras et al. (2007) (62) reported a high prevalence of metabolic syndrome in PLHIV, closely linked to insulin resistance and body fat redistribution.

With the advent of contemporary ART, metabolic toxicity has decreased, and several studies have reported no association between modern regimens and increased T2DM risk (33, 36, 37, 63, 64). However, controversies remain. Integrase strand transfer inhibitors (INSTIs), now the recommended first-line agents, have been consistently associated with greater weight gain compared with NNRTI- or PI-based regimens (7, 65, 66). Dolutegravir (DTG), in particular, has been linked to significant weight increases in both randomized trials (NAMSAL, ADVANCE) and observational cohorts (67–71), and some studies have reported progression to diabetes as a consequence of weight increases under DTG use (5). Spieler et al. (3) identified INSTIs as the only ART class independently associated with incident diabetes. Moreover, early-onset diabetes has been documented within 6–24 months of INSTI initiation in both clinical trials and observational cohorts (72–75). Overall, studies suggest INSTIs as a possible risk for PLHIV, either through a direct association to T2DM development or by aggravating other risk factors. In contrast, the 48 and 96-week results of the PASO DOBLE clinical trial demonstrated a distinct pattern, with participants receiving DTG plus lamivudine (3TC) experiencing significantly less weight gain compared with those on bictegravir (BIC)/emtricitabine (FTC), tenofovir alafenamide (TAF) (76), highlighting the discrepancies that still remain between studies. Altogether, current evidence supports weight gain as a major mediator of diabetes risk under INSTI-based regimens, while data suggesting weight-independent diabetogenic effects remain limited and inconsistent.

Nevertheless, associations with T2DM are not limited to INSTIs. In a South African case-control study, ritonavir- and lopinavir-based regimens were associated with strikingly high risks of diabetes, with odds ratios of 21 and 31, respectively (77). Similar associations with PIs have been reported elsewhere, often compounded by advanced disease markers such as low nadir CD4 counts and high peak HIV RNA levels (54, 78).

Taken together, ART-related risk of T2DM has evolved over time: earlier regimens conferred direct metabolic toxicity, while contemporary agents are safer but raise new concerns, particularly regarding weight gain under INSTI treatment. These findings emphasize the need to balance virological efficacy and metabolic health in the long-term management of HIV.

### 4.4 Other iatrogenic drivers

Beyond ART, other medications used in HIV care may also contribute to diabetes risk. Statins, frequently prescribed for cardiovascular prevention in PLHIV, have been associated with new-onset diabetes in the general population, particularly among those with pre-existing metabolic abnormalities. A subanalysis of the REPRIEVE trial found that pitavastatin use was associated with a clinically significant increase in diabetes incidence, but mainly among participants with three or more baseline metabolic risk factors (79). These findings suggest that statins may accelerate progression to diabetes in metabolically vulnerable PLHIV, underscoring the importance of careful risk stratification when initiating preventive therapies.

### 4.5 Emerging drivers: the gut microbiome

Gut dysbiosis has recently been proposed as a novel contributor to metabolic complications in PLHIV. Studies characterizing gut microbiota composition in PLHIV with metabolic syndrome have observed an imbalance of gut microbiota, with a decrease in abundance of bacterial species that included anti-inflammatory and metabolically beneficial bacteria, compared to PLHIV without metabolic syndrome (80, 81). Luo et al. (82) reported associations between specific gut bacterial taxa, metabolite signatures, and dysglycemia in HIV-positive and HIV-negative individuals, while Bar Ziv et al. (78) identified microbial profiles linked to hyperglycemia in PLHIV. Similarly, Shrivastav et al. (83) reviewed evidence for crosstalk between the gut bacteriome and virome in HIV and its potential role in metabolic disorders. Additionally, the combination of T2DM and HIV has been associated with lower gut microbiota diversity, increased tryptophan catabolism and higher levels of neopterin, a pro-inflammatory marker (84). These findings are consistent with broader evidence in non-HIV populations: Tang et al. (2022) described a bidirectional interplay between microbiome and viruses in periodontitis and T2DM, reinforcing the concept that microbial–viral interactions can influence metabolic health. Recent metabolomic data link metabolic-associated steatotic liver disease (MASLD) to bile acid and microbiota alterations. Chafino et al. (85) found elevated ursodeoxycholic acid (UDCA) and lipid species correlated with specific bacterial genera, delineating a gut–liver–metabolic axis that may contribute to insulin resistance. These findings reinforce that hepatic steatosis in HIV forms part of a broader immunometabolic network contributing to early-onset diabetes risk.

Additional studies support the relevance of gut-derived signals in HIV-related metabolic disease. Vestad et al. (86) demonstrated that plasma extracellular vesicles in PLHIV with T2DM correlated with markers of microbial translocation and cardiovascular risk, suggesting systemic consequences of dysbiosis. Interventional studies further highlight this axis: metformin, a first-line antidiabetic agent, has been shown to beneficially modulate gut microbiota and reduce inflammation in PLHIV (87, 88).

**Figure 1:**
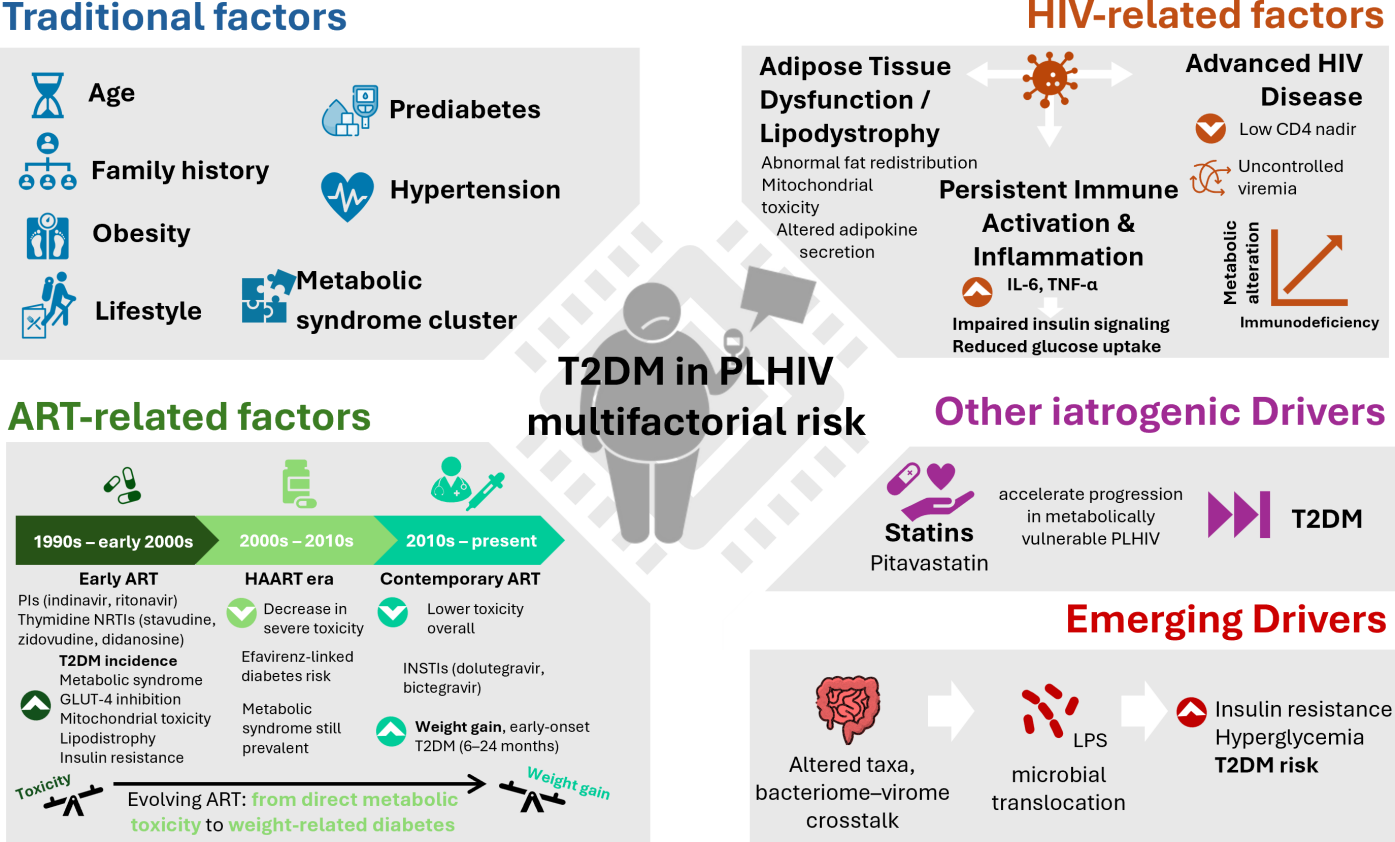
Drivers of type 2 diabetes mellitus in people living with HIV. T2DM in PLHIV arises from the interplay of multiple factors. Traditional metabolic risk factors (age, family history, obesity, lifestyle, hypertension, prediabetes, and clustering of metabolic syndrome components) mirror those in the general population. HIV-related factors, including chronic immune activation, inflammation (IL-6, TNF-α), adipose tissue dysfunction/lipodystrophy, and the legacy of advanced disease (low CD4 nadir, uncontrolled viremia), further impair glucose metabolism. ART-related factors have evolved over time: early regimens (protease inhibitors, thymidine NRTIs, efavirenz) induced direct metabolic toxicity, whereas contemporary integrase inhibitors (e.g., dolutegravir) are linked to weight gain and early-onset diabetes. Other iatrogenic drivers, such as statins, may accelerate progression in metabolically vulnerable PLHIV. Emerging drivers, particularly gut dysbiosis and microbial–viral interactions, promote microbial translocation, systemic inflammation, insulin resistance, and hyperglycemia. Together, these mechanisms highlight the multifactorial nature of diabetes risk in HIV. Abbreviations: ART, antiretroviral therapy; GLUT-4, glucose transporter type 4; IL-6, interleukin 6; INSTI, integrase strand transfer inhibitor; HAART, highly active antiretroviral therapy; LPS, lipopolysaccharide; NRTI, nucleoside reverse transcriptase inhibitor; NNRTI, non-nucleoside reverse transcriptase inhibitor; PI, protease inhibitor; PLHIV, people living with HIV; TNF-α, tumor necrosis factor; T2DM, type 2 diabetes mellitus.

Overall, the drivers of T2DM in PLHIV reflect the interplay of traditional metabolic factors (aging, obesity, lifestyle), HIV-related mechanisms (chronic inflammation, immune activation, adipose tissue dysfunction), and treatment-related exposures. Historical ART regimens, particularly first-generation PIs and thymidine analogues, left a legacy of metabolic toxicity, while contemporary therapies have reduced but not eliminated risk. The emergence of weight gain with INSTI-based regimens, especially DTG, has reintroduced concerns regarding early and long-term diabetes incidence. Additional iatrogenic factors, such as statin therapy, may further modulate individual risk. These interrelated drivers are summarized in **Figure 1**.

Taken together, these observations highlight the need for a multifaceted approach to diabetes prevention in HIV care: routine metabolic screening, proactive lifestyle interventions, and treatment decisions that balance virological efficacy with long-term metabolic safety.

## 5 Sex and gender disparities in type 2 diabetes among people living with HIV

### 5.1 Underrepresentation of women in HIV and T2DM research

Despite women and girls making up 53% of all PLHIV worldwide (89), they have consistently been underrepresented in HIV research and, consequently, in studies of T2DM in this population (90, 91). Historically, much of the metabolic evidence in HIV derived from male-dominated cohorts such as the Multicenter AIDS Cohort Study (MACS) (92), while the Women’s Interagency HIV Study (WIHS) (93) provided a critical but smaller counterpoint. This imbalance has limited the generalizability of findings, particularly regarding metabolic outcomes.

Sex-specific immunological differences highlight why this gap matters. In both adults (94) and children (95), female sex was overrepresented among elite controllers and non-progressors. Women living with HIV also demonstrate stronger immune control of other components of the human virome, such as Torque teno virus (96). Yet, paradoxically, immune activation and systemic inflammation are consistently higher in women than in men, independent of viral load, which may predispose to non-AIDS comorbidities (97, 98). Indeed, women, especially younger women, appear to carry an elevated risk of non-AIDS comorbidities (99, 100).

Patterns of female inclusion vary by geography and income setting. Curno et al. (90) showed that low- and middle-income countries (LMICs) are more likely to include women, reflecting regional HIV epidemiology. Recent T2DM-focused cohorts confirm this disparity: female participation was limited in Spain (12.7%) (36), the USA (21%) (3), and China (19.8%) (38), whereas representation was markedly higher in Ghana (78%) (101), South Africa (73.7%) (39) and Uganda (68.9%) (102). While such trends partly mirror epidemiology (in 2023, women accounted for 62% of new HIV infections in sub-Saharan Africa but only 27% outside this region (103)), they underscore the risk of producing evidence that is not fully generalizable. Moreover, regional differences in lifestyle, nutrition, and healthcare access directly affect metabolic comorbidities, including diabetes, making the underrepresentation of women in high-income settings particularly problematic.

### 5.2 Sex differences in weight gain and metabolic changes

One of the most consistent sex-dependent findings in HIV care relates to ART-associated weight changes. Women generally exhibit greater weight gain following ART initiation than men, across age groups and regimens (104). Lake et al. (66) identified female sex as a risk factor for weight gain after switching to INSTIs. In a pooled analysis of eight clinical trials in treatment-naïve PLHIV, Sax et al. (7) reported that women were more likely than men to experience >10% weight increase, with particularly strong associations among Black women. Similarly, Bourgi, Jenkings et al. (65) observed that female sex predicted substantial weight gain at both two and five years post-ART initiation, though a companion analysis by Bourgi, Rebeiro et al. (105) did not confirm sex as an independent predictor.

Evidence from randomized trials with balanced sex representation reinforces these observations. The NAMSAL and ADVANCE trials reported more pronounced weight gain in women starting DTG compared to men (68, 70, 71). Asundi et al. (106) found that INSTI use was associated with greater mean weight gain in women (11.0%, 95% CI: 5.2–16.8) and, importantly, with increased T2DM incidence (HR = 3.27, p = 0.01), independent of weight changes. Long-term analyses corroborate these findings: Lahiri et al. (107) reported that women experienced double the weight gain of men over five years after switching to INSTIs. Waist circumference, a surrogate for central adiposity, has also been shown to increase disproportionately in women. In analyses from the REPRIEVE trial, including more than 4,500 participants, both BMI and waist circumference increases were significantly more pronounced in women on INSTI-based regimens compared with men (108, 109).

Given the robust association between BMI and T2DM, the disproportionate weight and waist circumference increases observed in women may provide a mechanistic explanation for the higher diabetes prevalence reported in some cohorts.

### 5.3 Sex disparities in T2DM incidence and prevalence

Findings on the relationship between sex and T2DM incidence in PLHIV remain mixed. Collins et al. (110), in a large U.S. cohort of 2,300 adults aged ≥50 years with HIV under stable ART, reported a higher prevalence of T2DM in women compared to men (24% vs 17%), with diagnoses occurring during ongoing treatment. Birabaharan et al. (111) similarly reported higher diabetes prevalence in women (23%) than men (16%). Spieler et al. (3) also noted elevated risk in women, particularly Black women, although associations did not retain statistical significance after multivariable adjustment.

Conversely, several studies suggest higher diabetes incidence among men. Bratt et al. (33), in a Swedish population-based cohort, found greater T2DM incidence in men compared with women, while Lartey et al. (101) reported a threefold higher risk for men despite the cohort being predominantly female (78%). Supporting this, Geteneh et al. (46) found that male sex was independently associated with increased diabetes risk (adjusted odds ratio [aOR] = 4.29, 95% confidence interval [CI] = 1.08–17.04). Other cohorts, however, found no significant sex-based differences in incidence or glucose trajectories (64, 112).

Several mechanisms may underlie these inconsistencies. HIV has been linked to alterations in sex hormone-binding globulin (SHBG) and androgen levels, particularly in women, which may modulate diabetes risk. Higher androgen and SHBG levels are associated with lower diabetes risk in the general population, and women with HIV may therefore experience slower diabetes progression, though evidence remains inconclusive (113). The menopausal transition has also been identified as a critical life stage associated with increased visceral adiposity, insulin resistance, and metabolic complications in women with HIV, which may contribute to the higher diabetes prevalence observed in older female cohorts (114, 115). Finally, the degree of female representation within a study may also influence results, as suggested by Birabaharan et al. (111), complicating cross-cohort comparisons.

In summary, current evidence on sex differences in T2DM incidence and prevalence among PLHIV is inconsistent, with some cohorts reporting higher risk in women, others in men, and several showing no significant disparities. These conflicting findings suggest that epidemiological observations alone cannot fully explain sex-specific patterns of diabetes risk, highlighting the need to explore underlying biological mechanisms and gendered determinants, as discussed in the following section.

### 5.4 Potential mechanisms and gendered determinants

Biological explanations for sex-specific metabolic outcomes are beginning to emerge. Mechanistic studies suggest that INSTI-associated weight gain may involve dysregulation of appetite and energy homeostasis, possibly through reduced adipokine (leptin, adiponectin) release and altered melanocortin-4 receptor (MC4R) signaling (116, 117). *In vitro* and animal models have implicated DTG in mitochondrial dysfunction, increased lipid accumulation, and reduced thermogenesis, with more pronounced effects in females (118, 119). These findings may help explain the disproportionate weight gain and diabetes risk observed among women in clinical cohorts, although they remain preliminary and require validation at clinically relevant drug concentrations.

Beyond these pathways, chronic immune activation and systemic inflammation remain central mechanisms linking HIV and T2DM. Elevated levels of proinflammatory cytokines such as IL-6 and TNF-α, which persist even under effective ART, impair insulin signaling and promote glucose dysregulation (6). These immunological perturbations, together with ART-induced alterations in adipose tissue biology, may synergistically exacerbate metabolic vulnerability. The gut microbiome may also contribute to sex-specific metabolic outcomes. In the general population, microbiome composition shows measurable differences between men and women, which are thought to interact with sex hormones and metabolic regulation (120). Such differences could partly explain the inconsistent sex disparities in diabetes incidence observed among PLHIV. Although HIV-related microbiome studies have begun linking dysbiosis to metabolic alterations (78, 82, 83), sex-stratified analyses remain rare. Incorporating this dimension may help clarify whether microbial signatures modulate metabolic risk differently in men and women, particularly through pathways involving inflammation, adipose tissue function, and energy homeostasis.

**Figure 2:**
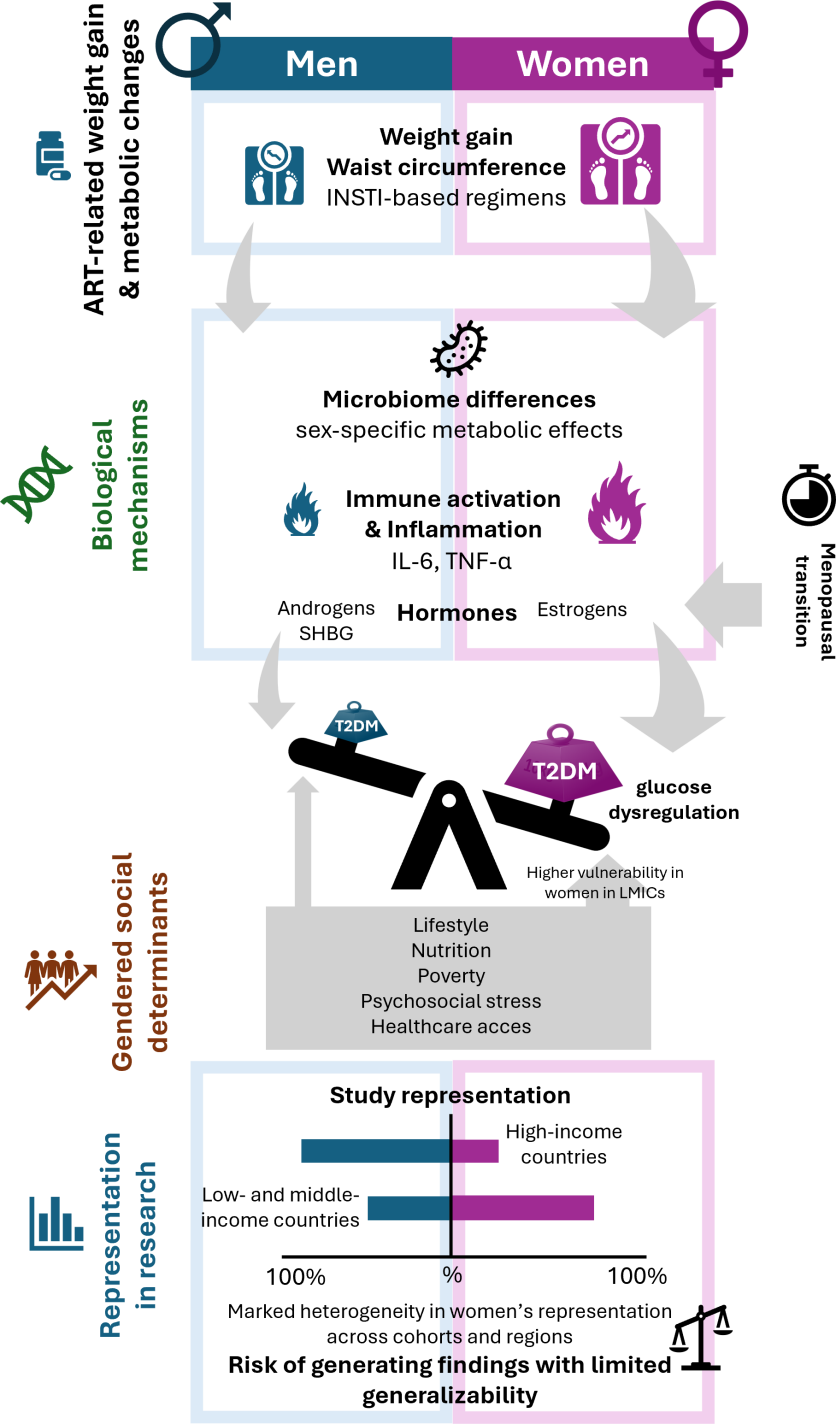
Sex disparities in type 2 diabetes mellitus among people living with HIV. Sex differences in T2DM risk arise from multiple interrelated domains. Representation in research remains uneven, with women often underrepresented in high-income settings and overrepresented in low- and middle-income countries, limiting the generalizability of findings. Biological mechanisms include sex-specific differences in immune activation, inflammation, hormonal milieu, and metabolic responses to antiretroviral therapy, leading to greater weight gain and central adiposity among women, particularly with integrase strand transfer inhibitors. Gendered social determinants, such as lifestyle, nutrition, poverty, psychosocial stress, and healthcare access, further modulate metabolic risk. Together, these interacting factors contribute to heterogeneous patterns of type 2 diabetes mellitus incidence and prevalence between men and women with HIV. Abbreviations: ART, antiretroviral therapy; IL-6, interleukin 6; INSTI, integrase strand transfer inhibitor; LMICs, low- and middle-income countries; PLHIV, people living with HIV; SHBG, sex hormone binding globulin; TNF-α, tumor necrosis factor; T2DM, type 2 diabetes mellitus.

Sex and gender disparities in T2DM among PLHIV therefore reflect a multifaceted interplay of underrepresentation in research, differential metabolic responses to ART, hormonal and immunological mechanisms, and social determinants of health. While many studies demonstrate greater ART-associated weight gain and, in some cohorts, higher diabetes prevalence among women, others report increased risk in men, highlighting persistent inconsistencies. What is clear, however, is that sex and gender remain critical, yet underexplored, dimensions of T2DM research in HIV. Ensuring adequate female representation, applying sex-stratified analyses, and incorporating intersectional perspectives (sex, race, age, menopause, geography, microbiome, and gendered social determinants) will be essential to clarify risk patterns and to optimize prevention and management strategies for all PLHIV. **Figure 2** provides a conceptual summary of sex and gender disparities in T2DM among PLHIV, integrating biological, social, and structural contributors.

## 6 Type 2 diabetes mellitus in adolescents and young adults living with HIV

Beyond ART regimen and sex, factors such as age, duration of HIV infection, and cumulative ART exposure also influence diabetes risk. Adolescents and young adults with perinatally acquired HIV (PHIV) represent a particularly vulnerable group. Globally, an estimated 1.4 million children (0–14 years) and 3.1 million adolescents and young adults (15–24 years) are living with HIV, the vast majority of whom were infected perinatally (89, 103). As ART initiation is recommended immediately after HIV diagnosis, this population is exposed to lifelong therapy and its potential long-term adverse effects.

In parallel, the past decade has witnessed a sharp rise in the global incidence and prevalence of T2DM in children and adolescents (121). PHIV youth not only share this general population risk but also face a higher likelihood of developing non-AIDS comorbidities compared to peers infected through other transmission routes (122). Moreover, these comorbidities occur at earlier ages and span a wide spectrum, including cardiovascular disease (123, 124), bone disorders (125), neurocognitive impairment (126), chronic kidney disease (127), and metabolic alterations (128, 129). This heightened risk reflects the combined effects of HIV infection in an immature immune system, chronic inflammation, and lifelong ART exposure.

### 6.1 Epidemiological evidence

A recent cohort study from the NA-ACCORD collaboration examined the incidence of non-AIDS comorbidities among 375 young adults with PHIV aged 18–30 years in North America. By age 30, the cumulative incidence of T2DM reached 19%, a strikingly high rate that underscores the importance of early metabolic screening in this population (127).

In sub-Saharan Africa, early metabolic abnormalities are increasingly documented. In Uganda, insulin resistance measured by HOMA-IR was already present in 6.7% of children and adolescents aged 10–16 years, with values rising significantly with age (130). In Tanzania, 13% of PHIV adolescents and young adults (aged 10–24 years) had impaired fasting glucose, with higher prevalence among males and those with central obesity (122). In Europe, Espiau et al. (131) described high prevalence of prediabetes and altered adipokine profiles (leptin, adiponectin, inflammatory markers) in Spanish children and adolescents with HIV-associated metabolic syndrome. Longitudinal U.S. data also highlight the risk of progression: between 1999 and 2019, 40% of PLHIV with prediabetes progressed to overt T2DM, compared with ~10% in the general population (132, 133).

Overall prevalence estimates vary widely, from 0% to 52% across pediatric cohorts (134), reflecting small sample sizes, heterogeneous definitions, and limited follow-up. Nevertheless, the evidence suggests a progressive rise in prediabetes and diabetes risk as PHIV populations age, with some cohorts indicating that future incidence may surpass that observed in adults with HIV acquired later in life (122).

### 6.2 Pathophysiological, developmental, and social considerations

Beyond insulin resistance and adipose dysfunction, liver involvement is increasingly recognized as a metabolic driver of dysglycemia in adolescents and young adults with perinatally acquired HIV. Studies using non-invasive imaging have shown a higher prevalence of metabolic-associated steatotic liver disease (MASLD) in this population compared with HIV-negative peers, even after adjustment for BMI and lifestyle factors. Carrasco et al. (128) and Rose et al. (135) reported that hepatic steatosis affected up to one third of youth with long-term ART exposure and was inversely associated with CD4 count and duration of viral suppression, suggesting HIV- and ART-related metabolic disturbances beyond obesity.

Recent mechanistic data indicate that bile acid metabolism and gut microbiota composition may mediate these hepatic and glycemic alterations. In a metabolomic study of adolescents with HIV, Chafino et al. (85) identified elevated circulating UDCA and lipid species that correlated with specific microbial genera (*Blautia*, *Collinsella*, *Faecalibacterium*), delineating a gut–liver–metabolic axis potentially linking steatosis and insulin resistance. The combined model including UDCA, triglyceride species, and the hepatic steatosis index improved discrimination of MASLD cases, supporting their role as candidate biomarkers of metabolic dysfunction.

These findings reinforce the concept that hepatic steatosis in young adults with PHIV is part of a broader immunometabolic spectrum contributing to insulin resistance and early-onset T2DM. Integrating liver assessment and bile acid–microbiota markers into metabolic screening may enhance early detection of dysglycemia and guide preventive strategies in this vulnerable population.

Adolescence is a developmental period characterized by physiologic insulin resistance, which in PHIV may be exacerbated by chronic immune activation, long-term ART exposure, and adipose tissue alterations. Espiau et al. (131) linked HIV-associated metabolic syndrome in youth to dysregulated adipokine signaling, while Blázquez et al. (136) reported increasing rates of insulin resistance among older adolescents and young adults with PHIV.

Puberty itself compounds this vulnerability. Reduced insulin sensitivity during pubertal transition may interact with HIV- and ART-related factors, amplifying long-term metabolic risk (123). When diabetes develops at a young age, progression is typically more aggressive, with accelerated decline in β-cell function and earlier onset of complications (121). Moreover, diagnostic challenges exist, as HbA1c thresholds validated in adults are less reliable in pediatric populations, potentially leading to under- or misdiagnosis (137).

**Figure 3:**
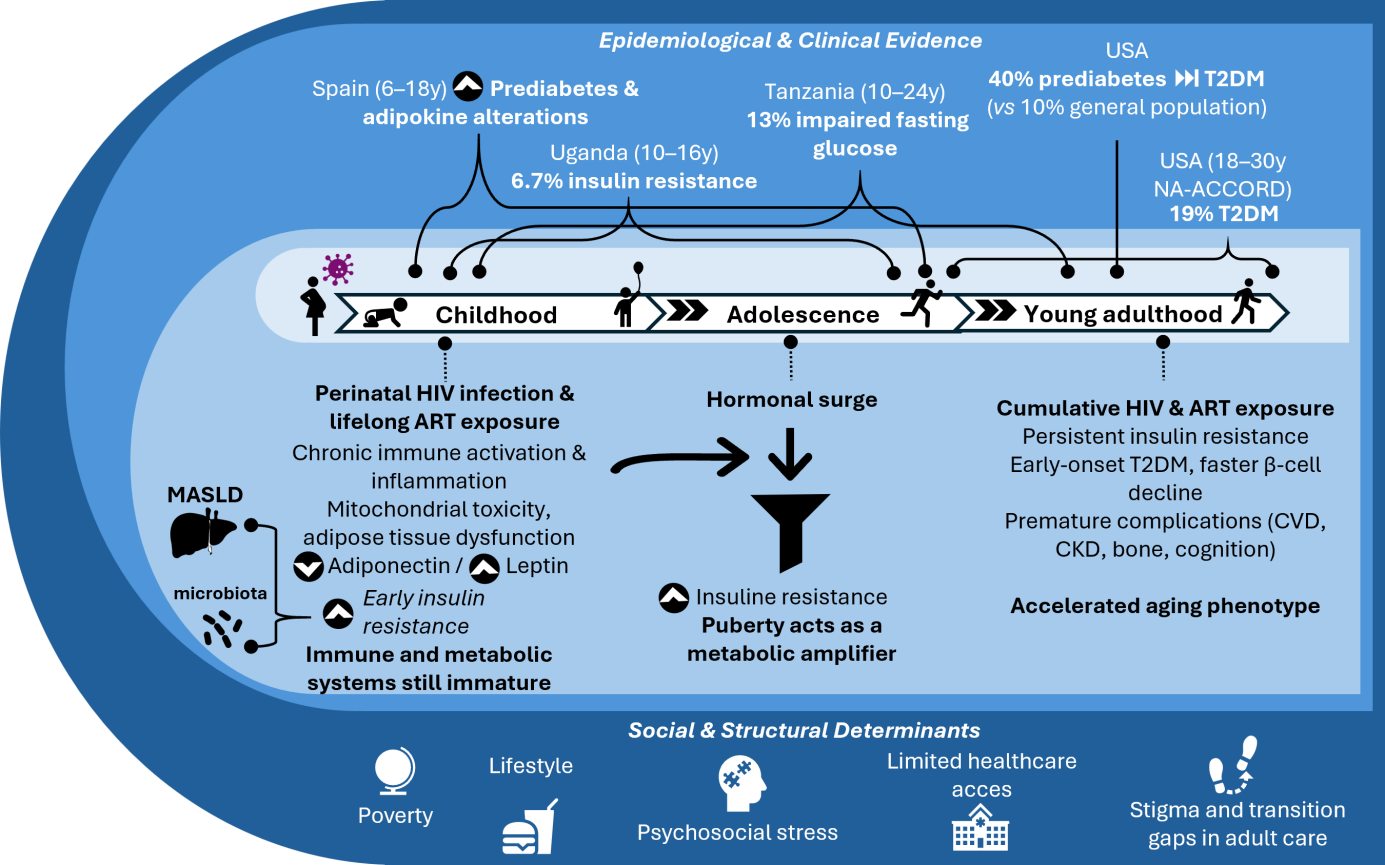
Lifelong metabolic vulnerability in adolescents and young adults with perinatally acquired HIV. Children and adolescents with perinatal HIV infection are exposed to lifelong ART, chronic inflammation, and adipose tissue dysfunction from early life. During puberty, hormonal changes act as a metabolic amplifier, enhancing insulin resistance and accelerating β-cell stress. Hepatic steatosis and early gut microbiota further exacerbate metabolic dysregulation, promoting insulin resistance and liver-driven inflammation. Cumulative exposure to HIV and ART during young adulthood contributes to persistent dysglycemia, early-onset T2DM, and premature comorbidities such as cardiovascular, kidney, bone, and cognitive complications. Epidemiological evidence across continents highlights the progressive increase in insulin resistance, impaired fasting glucose, and T2DM with age. Social and structural determinants further exacerbate risk in this population. Abbreviations: ART, antiretroviral therapy; CKD, chronic kidney disease; CVD, cardiovascular disease; MASLD, metabolic-associated steatotic liver disease; NA-ACCORD, North American AIDS Cohort Collaboration on Research and Design; PHIV, perinatally HIV-infected; T2DM, type 2 diabetes mellitus.

In addition to biological and developmental mechanisms, social and structural determinants significantly shape metabolic outcomes in PHIV populations. A South African study by Van Wyk and Roomaney (138) showed that non-communicable conditions, including T2DM, were closely linked to poverty, food insecurity, lifestyle, and psychosocial stress. These findings reflect a syndemic context in which biological risk factors are amplified by social vulnerability, highlighting the importance of integrated prevention strategies. **Figure 3** summarizes the life-course trajectory of metabolic vulnerability PHIV youth, integrating biological, developmental, epidemiological, and social determinants that contribute to the early onset of T2DM.

### 6.3 Implications for long-term risk and follow-up

Although T2DM remains relatively uncommon in pediatric HIV cohorts, the combination of early metabolic abnormalities, lifelong ART exposure, and developmental vulnerabilities suggests a trajectory of increasing risk into adulthood. Early and repeated metabolic screening is therefore critical, particularly for adolescents transitioning to adult HIV care. As previously mentioned, some of the current diagnostic methods used are unreliable in pediatric populations and thus identifying predictive biomarkers in PHIV populations could help stratify risk and guide preventive interventions. Given that youth-onset T2DM is associated with faster progression and earlier complications than adult-onset disease, proactive monitoring and preventive strategies are essential to mitigate long-term morbidity in this vulnerable group.

When considering long-term implications and follow-up among pediatric HIV cohorts, the transition from pediatric to adult care is critical to maintain care and treatment continuity. Challenges such as novelty of providers and environment, communication gaps between teams, stigma and lack of support often result in patients falling out of care, interruption of treatment, loss of viral suppression and disease progression (139). This poses another risk for youths living with HIV that further increases danger of future T2DM development, and action towards ensuring a smoother healthcare transition should be taken accordingly.

Given the high prevalence of early insulin resistance and prediabetes in adolescents and young adults with PHIV, current diabetes screening recommendations based on adult populations may be insufficient for this group. Earlier initiation of screening and more frequent metabolic monitoring during adolescence and the transition to adult care may be warranted.

## 7 Diagnosis, predictive biomarkers, and management of type 2 diabetes mellitus in people living with HIV: challenges and opportunities

### 7.1 Diagnosis and screening

The screening for T2DM and other metabolic comorbidities in PLHIV is strongly recommended by consensus projects and clinical guidelines (140–142). Standard diagnostic approaches include fasting plasma glucose (FPG), oral glucose tolerance test (OGTT), and HbA1c. According to the American Diabetes Association (121), T2DM is diagnosed when FPG is ≥ 126 mg/dL (7.0 mmol/L), or 2-hour plasma glucose during a 75 g OGTT is ≥ 200 mg/dL (11.1 mmol/L), or HbA1c is ≥ 6.5% (48 mmol/mol). In the absence of unequivocal hyperglycemia, diagnosis requires two abnormal results from different tests obtained at the same time, or the same test repeated on two separate occasions. Additionally, a random plasma glucose ≥ 200 mg/dL (11.1 mmol/L) in the presence of classic symptoms of hyperglycemia is also diagnostic.

However, the use of HbA1c is limited in many resource-constrained settings, where laboratory-based testing may not be routinely available. Although validated point-of-care HbA1c devices have been developed (143, 144), their high cost and limited accessibility restrict widespread implementation in sub-Saharan Africa and other low-resource regions. Even when available, HbA1c presents important limitations in PLHIV. In a prospective study, Kim et al. (145) demonstrated that HbA1c underestimated mean blood glucose by approximately 30 mg/dL in adults with HIV, an effect strongly linked to macrocytosis and exposure to thymidine analogue NRTIs such as abacavir, zidovudine, and lamivudine. In contrast, fructosamine provided a more accurate estimate of glycemia and was not affected by ART-related hematologic changes.

In light of these concerns, multiple clinical guidelines—including those from the American Diabetes Association, the HIV Medicine Association, the Australian HIV guidelines, and the European AIDS Clinical Society—recommend prioritizing FPG, with or without OGTT, as the preferred diagnostic approach in PLHIV (141, 146).

In addition to single tests, clinical risk scores have been evaluated for predictive utility. In a prospective analysis of the Swiss HIV Cohort, Blondet et al. (147) found that three established diabetes risk scores, FINDRISC2, the Swiss Diabetes Association score, and the Balkau score, showed strong discriminatory capacity, with area under the curve (AUC) values of 0.81 and negative predictive values >98%. These findings suggest that risk scores may help identify low-risk individuals in whom intensive screening may be unnecessary, though HIV-specific models are still needed to capture unique ART- and inflammation-related risks.

Early and repeated screening is particularly warranted in high-risk groups, including PHIV youth and older adults, where cumulative ART exposure and chronic inflammation may amplify metabolic vulnerability. Nonetheless, no single biomarker provides a universally reliable standard for diagnosing T2DM in this population. Evidence questioning HbA1c accuracy remains mixed, and existing alternatives, such as fructosamine, are not yet widely adopted in clinical practice. This highlights the ongoing need for research to establish optimal diagnostic thresholds and identify reliable biomarkers tailored to the specific metabolic and hematologic context of PLHIV.

### 7.2 Predictive biomarkers

To complicate matters further, there is no internationally agreed diagnostic or predictive biomarker for T2DM in PLHIV.

Fasting glucose levels at HIV diagnosis have been shown to predict later development of T2DM. Brown et al. (148) reported that individuals with higher baseline glucose at HIV diagnosis had a significantly greater risk of progression to diabetes. Consistent with this, preliminary results from the Spanish DIAVIH study (Tarancon-Diez et al., GeSIDA, 2025) confirmed the predictive value of early metabolic markers. At HIV diagnosis, individuals who later developed T2DM had significantly higher baseline fasting glucose and triglycerides compared with matched controls. Notably, more than half of the cases already exhibited fasting glucose >100 mg/dL—meeting criteria for prediabetes—as early as four years before diabetes diagnosis. These findings emphasize the need for systematic early metabolic monitoring in PLHIV to identify high-risk individuals years before overt T2DM develops.

On the other hand, C-peptide, although extensively studied in the general population as a marker to distinguish between diabetes types and to assess β-cell function, remains largely unexplored in the context of T2DM in PLHIV. Elevated C-peptide levels have been proposed as predictors of dyslipidemia in PLHIV (149). In non-HIV populations, both fasting and stimulated C-peptide levels are widely used to estimate endogenous insulin secretion and carry prognostic value in determining insulin dependency and guiding therapeutic strategies (150). Incorporating C-peptide into future HIV cohorts could therefore help refine metabolic risk stratification.

Beyond classical glycemic measures, several studies have identified immune and inflammatory markers as potential predictors. Elevated levels of high-sensitivity C-reactive protein (hsCRP) and IL-6 predicted incident T2DM in PLHIV independently of FPG, BMI, and age in a five-year prospective study (148, 151). Similarly, immune senescence markers, such as terminally differentiated (CD45RA^+^CD27^-^) and senescent (CD28^-^) CD4 T-cells, were associated with incident diabetes in the Veterans Aging Cohort Study (152). Consistent findings were reported by Chukwuanukwu et al. (153), who described elevated IL-6 and CRP, together with reduced CD4 counts in PLHIV with T2DM, supporting a role for chronic immune activation and low-grade inflammation in diabetes pathogenesis. By contrast, Bernardino et al. (154) found no association between a panel of monocyte and aging-related biomarkers and diabetes risk, likely due to the small number of incident cases, underscoring the need for larger, adequately powered studies. Beyond classical inflammatory biomarkers, hepatic comorbidities also act as predictors. In a longitudinal study, Han et al. (155) found that baseline non-alcoholic fatty liver disease (NAFLD), with or without non-alcoholic steatohepatitis (NASH), was associated with higher diabetes incidence, particularly when combined with liver fibrosis. These findings highlight the contribution of liver dysfunction to metabolic risk in PLHIV.

Beyond inflammation, other emerging biomarkers have been proposed. Circulating microRNAs (miRNAs) such as miR-126, miR-146a, miR-29, and miR-375 are associated with insulin resistance and β-cell dysfunction in non-HIV populations (156), though validation in PLHIV cohorts is still lacking. These stable, easily detectable non-coding RNAs in plasma may serve as useful predictive and diagnostic tools for metabolic monitoring.

Omics-based approaches are providing new insights. Gonzalez-Izundegui et al. (157) identified branched-chain amino acids and dimethylguanidino valeric acid as metabolic signatures associated with diabetes risk. In addition, microbiota-derived metabolites may play a role: Jia et al. (158) reported compartment-specific associations in women with HIV, with plasma kynurenate positively linked to diabetes risk, while fecal levels showed inverse associations. Gut microbiome composition itself differs between men and women and has been linked to insulin resistance and prediabetes (159), suggesting a role for microbiota-driven biomarkers in explaining sex-specific metabolic outcomes in PLHIV. Immunometabolic markers may also be relevant: Butterfield et al. (160) showed increased GLUT1 expression and glycolytic activity in CD4 T-cells from HIV-positive women with T2DM, suggesting a mechanistic link between immune cell metabolism and diabetes.

### 7.3 Therapeutic approaches

Pharmacological treatment of T2DM in PLHIV generally mirrors that in the general population, with metformin as the first-line agent. Beyond glucose control, metformin may reduce CD4 T-cell exhaustion (161), inhibit mTOR signaling (162), and modulate gut microbiota diversity (87, 163). These immunometabolic effects suggest potential benefits for HIV reservoir control (164), although findings are mixed: Rezaei et al. (165) reported increased HIV transcription with metformin via CREB phosphorylation. Moreover, cognitive benefits observed in the general population were not confirmed in a small PLHIV cohort (166).

When metformin alone is insufficient, insulin remains safe and effective. In recent years, glucagon-like peptide-1 receptor agonists (GLP-1RAs) have shown particular promise. Case reports describe improved glycemic control and weight reduction with exenatide, liraglutide, and dulaglutide (167–169). Observational data confirm BMI reduction in PLHIV with diabetes (170). Stronger evidence comes from a phase 2b randomized trial in PLHIV with lipohypertrophy, where semaglutide reduced visceral fat by 30% and total body fat by nearly 19% (171). Despite these benefits, GLP-1RAs remain underused in practice: in an Australian cohort, only 19% of eligible PLHIV with diabetes received them (172).

In pediatric and adolescent populations, incretin-based therapies are also gaining relevance. Trials of liraglutide and semaglutide in children and adolescents with obesity or T2DM (173–175) demonstrated significant improvements in BMI, HbA1c, and hepatic steatosis markers. Rose et al. (176) further reported reductions in hepatic fat and improved lipid profiles after switching to DTG-based ART, suggesting that optimizing both ART and metabolic therapy may yield synergistic benefits.

### 7.4 Lifestyle and structural barriers

Lifestyle modification is fundamental to diabetes prevention and treatment, yet physical activity and dietary adherence are often insufficient among PLHIV (177). Beyond individual behaviors, structural determinants remain major obstacles. Qualitative studies from Ethiopia, Zimbabwe, and Côte d’Ivoire highlight fragmented care between HIV and non-communicable disease (NCD) services, medication shortages, healthcare-related stigma, and financial barriers such as lack of insurance (178–181).

Such gaps translate into suboptimal outcomes: in a Mexico City tertiary center, although 94% of PLHIV with diabetes achieved viral suppression, fewer than half met glycemic targets (182). These findings underscore the importance of integrated HIV–NCD services, particularly in resource-limited settings. Strengthening primary healthcare, reducing stigma, and ensuring access to affordable antidiabetic therapies will be essential to address the dual burden of HIV and T2DM.

**Figure 4:**
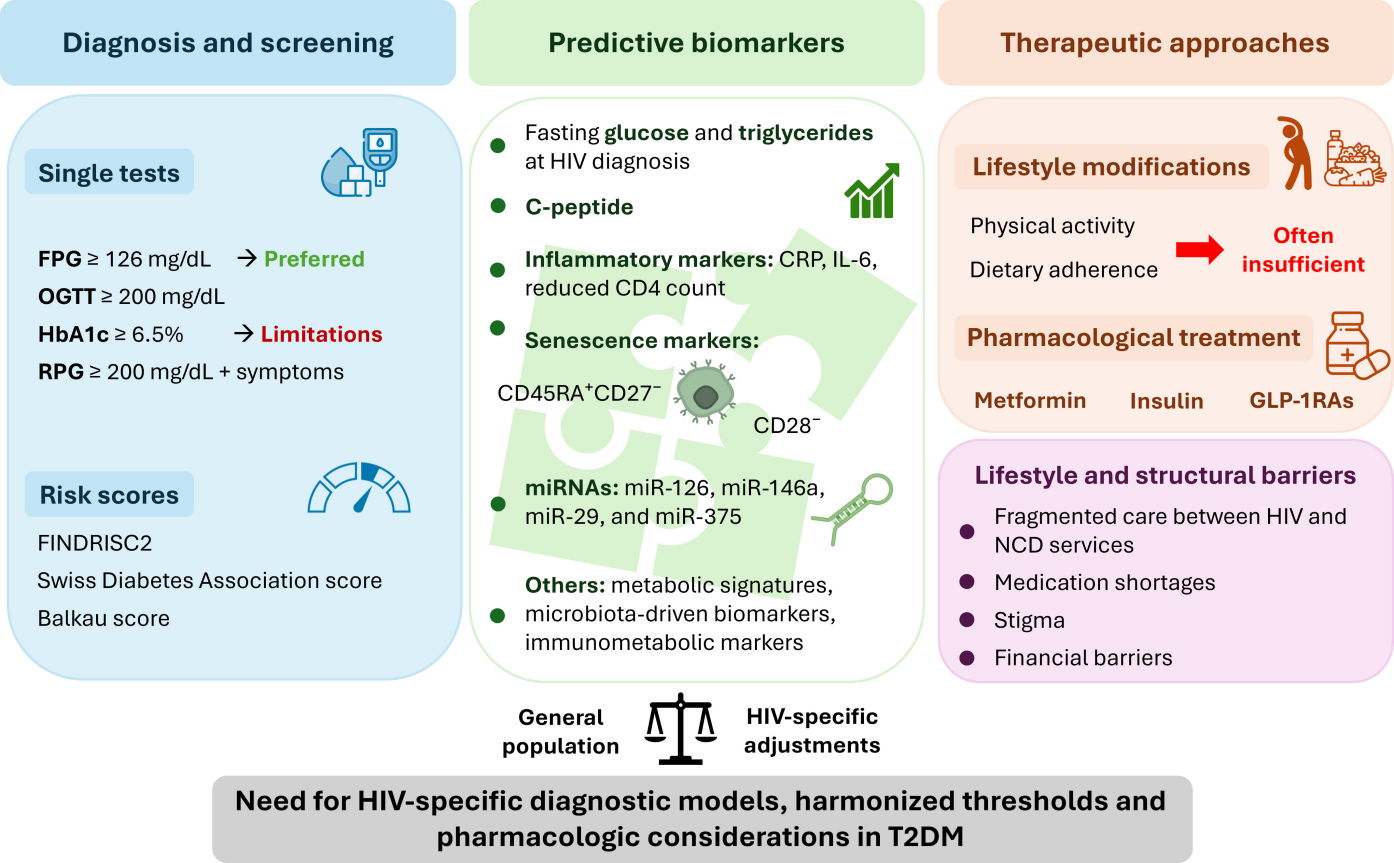
Diagnosis, predictive biomarkers, and management of type 2 diabetes mellitus in people living with HIV. This schematic summarizes the key domains involved in the diagnosis and management of T2DM among PLHIV. Diagnostic screening primarily relies on fasting plasma glucose and oral glucose tolerance test, while glycated hemoglobin (HbA1c) may underestimate glycemia due to ART-related hematologic changes. Random plasma glucose (RPG) ≥200 mg/dL in the presence of classic hyperglycemia symptoms is also diagnostic. Risk prediction tools such as FINDRISC2, the Swiss Diabetes Association score, and the Balkau score have shown good discriminatory capacity for identifying individuals at low or high risk of diabetes, although none are specifically validated for PLHIV. Predictive biomarkers include metabolic markers (glucose, triglycerides, C-peptide), inflammatory markers (C-reactive protein, IL-6), immune senescence markers (CD45RA^+^CD27^-^, CD28^-^), and emerging omics signatures such as microRNAs (miR-126, miR-146a, miR-29, miR-375) and metabolic profiles. Therapeutic approaches combine lifestyle modifications, often insufficient, with pharmacologic treatment using metformin, insulin, and glucagon-like peptide-1 receptor agonists. Structural barriers including fragmented care between HIV and non-communicable disease services, medication shortages, stigma, and financial constraints remain major challenges. Collectively, these factors highlight the need for HIV-specific diagnostic models, harmonized thresholds, and pharmacologic considerations adapted to the unique metabolic and immunologic context of PLHIV. Abbreviations: ART, antiretroviral therapy; CRP, C-reactive protein; FINDRISC2, Finnish Diabetes Risk Score; FPG, fasting plasma glucose; GLP-1RA, glucagon-like peptide-1 receptor agonist; HbA1c, glycated hemoglobin; IL-6, interleukin-6; miRNA, microRNA; NCD, non-communicable disease; OGTT, oral glucose tolerance test; PLHIV, people living with HIV; RPG, random plasma glucose; T2DM, type 2 diabetes mellitus.

Collectively, these diagnostic, biological, and structural challenges are summarized in **Figure 4**, which outlines the interconnected domains of screening, predictive biomarkers, therapeutic strategies, and healthcare barriers shaping the management of T2DM in PLHIV.

## 8 Conclusions and future perspectives

The global burden of T2DM among PLHIV is increasing, reflecting the convergence of traditional metabolic risk factors, HIV-related immune and inflammatory pathways, and the long-term effects of ART. Early diagnosis is critical, as delayed recognition of glycemic abnormalities may accelerate the onset of complications and worsen long-term outcomes. In this context, there is an urgent need for robust, predictive biomarkers that can complement or even surpass standard diagnostic tools, enabling timely identification of high-risk individuals and guiding personalized prevention strategies.

Future research should prioritize the validation of immune-inflammatory markers, liver- and adipose-related indicators, and emerging candidates such as circulating microRNAs and microbiota-derived metabolites. Integrating these biomarkers into risk stratification models tailored to HIV-specific pathophysiology will be essential to move beyond a one-size-fits-all approach.

At the same time, therapeutic innovation is advancing. In addition to metformin, which may exert immunomodulatory and microbiome-modifying effects, newer agents such as GLP-1RAs inhibitors hold promise for improving both glycemic control and cardiometabolic outcomes in PLHIV. Yet, their underuse in clinical practice underscores the need for more inclusive trials and updated guideline recommendations that address the dual burden of HIV and T2DM.

Another emerging challenge is polypharmacy, as PLHIV with T2DM frequently require multiple medications for metabolic, cardiovascular, and other comorbid conditions in addition to lifelong ART. This therapeutic complexity increases the risk of drug-drug interactions, cumulative toxicity, and reduced adherence (183–185). Future strategies should emphasize integrated care models, systematic medication reconciliation, and the development of predictive tools to anticipate and mitigate interactions, ensuring safe and effective long-term management.

Finally, achieving progress in this field requires addressing structural barriers. Health systems must strengthen the integration of HIV and NCD services, particularly in resource-limited settings where laboratory diagnostics and access to novel therapies remain constrained. Incorporating lifestyle interventions, sex- and age-specific considerations, and intersectional social determinants of health will be equally important to reduce disparities.

In summary, advancing the care of PLHIV at risk for or living with T2DM will demand a multidimensional agenda: early diagnosis, biomarker-driven risk prediction, therapeutic innovation, integrated care pathways, and health policy reforms. By aligning clinical, biological, and structural perspectives, future strategies can move toward precision medicine approaches that improve metabolic outcomes and quality of life for all PLHIV.

Ultimately, integrating metabolic risk management into HIV care, validating HIV-specific biomarkers, and ensuring equitable access to novel therapies should be central priorities to mitigate the rising burden of T2DM in this population.
